# Molecular Surveillance for Lymphoproliferative Disease Virus in Wild Turkeys (*Meleagris gallopavo*) from the Eastern United States

**DOI:** 10.1371/journal.pone.0122644

**Published:** 2015-04-21

**Authors:** Jesse M. Thomas, Andrew B. Allison, Edward C. Holmes, Jamie E. Phillips, Elizabeth M. Bunting, Michael J. Yabsley, Justin D. Brown

**Affiliations:** 1 Southeastern Cooperative Wildlife Disease Study, Department of Population Health, 589 D.W. Brooks Drive, Wildlife Health Building, College of Veterinary Medicine, The University of Georgia, Athens, Georgia, United States of America; 2 Daniel B. Warnell School of Forestry and Natural Resources, The University of Georgia, Athens, Georgia, United States of America; 3 Baker Institute for Animal Health, Department of Microbiology and Immunology, College of Veterinary Medicine, Cornell University, Ithaca, New York, United States of America; 4 Marie Bashir Institute for Infectious Diseases and Biosecurity, Charles Perkins Centre, School of Biological Sciences and Sydney Medical School, University of Sydney, Sydney, New South Wales, Australia; 5 Animal Health Diagnostic Center, College of Veterinary Medicine, Cornell University, Ithaca, New York, United States of America; Linneaus University, SWEDEN

## Abstract

Lymphoproliferative disease virus (LPDV) is a poorly understood, oncogenic avian retrovirus of domestic turkeys that has historically been restricted to Europe and Israel. However, a recent study reported LPDV in multiple wild turkey diagnostic cases from throughout the eastern United States of America (USA). To better understand the distribution of LPDV in the eastern USA, we surveyed 1,164 reportedly asymptomatic hunter-harvested wild turkeys from 17 states for the presence of LPDV proviral DNA by PCR. In total, 564/1,164 (47%) turkeys were positive for LPDV. Wild turkeys from each state had a relatively high prevalence of LPDV, although statewide prevalence varied from 26 to 83%. Phylogenetic analysis revealed two major clades of LPDV in the USA, although one was at a low frequency suggesting restricted transmission, as well as significant clustering by state of isolation. To determine the best tissue to target for diagnostic purposes, liver, spleen, and bone marrow were tested from a subset of 15 hunter-harvested wild turkeys and 20 wild turkey diagnostic cases. Overall, bone marrow provided the highest level of detection for both hunter-harvested turkeys and diagnostic cases. The sensitivity of LPDV detection between tissues was not significantly different for diagnostic cases, but was for hunter-harvested birds. These results indicate that LPDV infection is common and widespread in wild turkey populations throughout the eastern USA, even without overt signs of disease.

## Introduction

Neoplastic diseases of gallinaceous poultry can be non-infectious (i.e., spontaneous) or induced by viruses. Non-infectious neoplasms in poultry are sporadic in occurrence, typically affect older birds, and are of minor economic significance to the poultry industry [1]. Viral-induced neoplasms in gallinaceous poultry are most commonly caused by one of three viruses: (i) Marek’s disease virus type 1 (MDV), (ii) avian leukosis virus (ALV), and (iii) reticuloendotheliosis virus (REV).

Marek’s disease virus, also known as gallid herpesvirus 2 (genus *Mardivirus*, subfamily *Alphaherpesvirinae*, family *Herpesviridae*), produces lymphoproliferative disease in domestic chickens and, occasionally, in domestic turkeys, quail, and pheasants [2]. The virus is highly contagious and can be transmitted horizontally through the inhalation of contaminated dust in poultry houses [1]. Marek’s disease virus is ubiquitous in domestic chicken populations throughout the world and is a significant source of economic losses due to mortality, condemnation, decreased production, and/or costs associated with control [1]. Since the introduction of vaccination of commercial poultry in the early 1970’s, mortality associated with MDV has dropped significantly; however, sporadic outbreaks still occur as new virulent viral strains emerge for which vaccines are not fully protective [1].

Avian leukosis virus (genus *Alpharetrovirus*, subfamily *Orthoretrovirinae*, family *Retroviridae*) produces a wide-variety of hemopoietic and connective tissue tumors in domestic chickens, but the most commonly observed neoplasms are lymphoid leukosis and myeloid leukosis [3]. Transmission of ALV can occur either vertically (from infected hen to offspring via the egg) [4] or horizontally through direct or indirect exposure [3–5]. Similar to MDV, ALV is ubiquitous in domestic chicken populations worldwide; however, the incidence of neoplasms or other overt signs of disease in infected flocks is typically low (1–2%) [3]. Despite the relatively low incidence of morbidity and mortality, ALV is still considered to have significant economic impacts on poultry due to losses associated with mortality, condemnation, and reduced production [3].

Reticuloendotheliosis virus (genus *Gammaretrovirus*, subfamily *Orthoretrovirinae*, family *Retroviridae*) produces several neoplastic and non-neoplastic disease syndromes in birds, including runting syndrome, chronic lymphoid neoplasms, and acute reticulum cell neoplasia [6]. As opposed to ALV and MDV, which are predominately pathogens of chickens, REV can cause disease in a variety of wild and domestic avian species, including domestic and wild turkeys, chickens, waterfowl, and upland game birds [6–9]. Similar to ALV, REV can be transmitted vertically or horizontally through direct or indirect routes [9]. Although REV infection is widespread and common in domestic poultry in several countries, overt disease, including tumors or runting syndrome, is uncommon and most infections are silent [10]. Consequently, the economic importance of REV for the poultry industry is overall considered to be less than MDV or ALV.

A rare neoplastic syndrome of domestic turkeys called lymphoproliferative disease is caused by a distinct retrovirus, lymphoproliferative disease virus (LPDV) (tentatively classified in the genus *Alpharetrovirus*, subfamily *Orthoretrovirinae*, family *Retroviridae*) [11]. Outbreaks of LPDV in domestic turkeys have been extremely rare and only reported in a few European countries and Israel [12, 13]. The characteristic lesion of lymphoproliferative disease is the infiltration of pleomorphic lymphoid cells (including lymphocytes, lymphoblasts, reticulum cells, and plasma cells) into multiple visceral organs and tissues. Mortality during LPDV outbreaks has been relatively low and young poults, between 8 and 18 weeks of age, have been the most severely affected [12, 14]. The natural routes of LPDV transmission in domestic turkeys are not currently known. Experimentally, LPDV can be transmitted horizontally between in-contact turkey poults, although the primary mechanism of infection or spread within a flock has not been identified [15]. Additionally, it is unknown if LPDV can be transmitted vertically. To date, LPDV has not been cultured *in vitro* and this has significantly limited surveillance, diagnostics, and research efforts to better understand the virus.

In domestic poultry, retroviral-induced neoplasms are inherently challenging to study as different viruses can induce similar tumors and lesions, individual viral species can produce a variety of neoplastic syndromes, and infection in domestic poultry may be widespread in the absence of overt disease. These challenges are compounded in wild birds by a lack of surveillance data on the incidence or prevalence of retroviruses in wild populations, along with details of their epidemiology. In general, neoplasia in wild birds is rare [9,16]. However, lymphoproliferative neoplasms have sporadically been reported in a variety of wild and captive exotic galliforms, including wild turkeys (*Meleagris gallopavo*), ring-necked pheasants (*Phasianus colchicus*), greater prairie chickens (*Tympanuchus cupido*), Attwater’s prairie chickens (*Tympanuchus cupido attwateri*), common peafowl (*Pavo cristatus*), and Japanese quail (*Coturnix japonica*) [8–9,17–24]. While no causative virus was identified from many of these cases, REV was detected in multiple birds diagnosed with lymphoid tumors [8,22–24].

Historically, LPDV had not been reported from wild birds or domestic poultry in North America; however, the virus was first identified in an adult wild turkey with lymphoproliferative disease from Arkansas in 2009 [11]. Following this initial detection, wild turkey carcasses submitted to diagnostic laboratories from throughout the eastern United States of America (USA) were screened for LPDV and the virus was identified in 17 additional states [11]. Interestingly, of the 39 LPDV positive birds in which tissues were examined microscopically in this study, only 6 (15%) had evidence of lymphoproliferative disease. Additionally, in this same study, a high prevalence (45%; 33/74) of LPDV was detected in liver samples collected from reportedly asymptomatic hunter-harvested wild turkeys from South Carolina. Collectively, these preliminary data suggest that LPDV infection may be common in North American wild turkeys, even in the absence of clinical signs.

The goal of this current study was to undertake a more extensive survey of the prevalence and phylogenetic structure of LPDV in wild, asymptomatic wild turkeys from throughout the eastern USA. To this end, we sampled liver, spleen, or bone marrow from 1,164 hunter-harvested wild turkeys from 17 states for LPDV using PCR, and performed phylogenetic analysis on 185 sequences of the p31-capsid (CA) genes. Additionally, in an effort to facilitate future surveillance efforts, we tested three tissues (liver, spleen, and bone marrow) from a subset of wild turkeys to determine the most appropriate diagnostic sample for detecting LPDV.

## Methods

### Sample Collection

**Fig 1:**
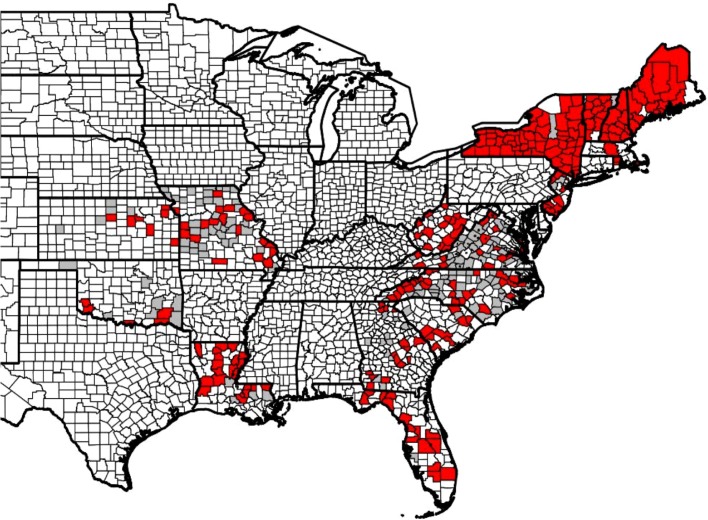
Geographic distribution of wild turkey samples from 17 states by county that tested positive (red) or negative (gray) for LPDV proviral DNA.

During the spring 2011, fall 2012, and spring 2013 wild turkey hunting seasons, samples were collected from hunter-harvested wild turkeys in 417 counties from 17 states (Fig 1). Tissue samples were obtained from hunter-harvested turkeys either directly by state biologists or by having hunters collect the samples and send them to their respective state agencies. Sample collection protocols were approved by the University of Georgia’s Institutional Animal Care and Use Committee (#A2007-10-186). Based on previous diagnostic testing of a variety of tissues from LPDV-infected wild turkeys [11], liver, spleen, and bone marrow were selected as target tissues for this study. At least one of the three tissues was collected from each hunter-harvested turkey included in this study; however, the specific tissue(s) varied between states based on expertise of personnel, availability of resources, and existence of ongoing sampling efforts by individual agencies. Each tissue sample was collected and stored individually in Whirl-pak (spleen and liver) or Ziploc (bone marrow) bags. Where possible, efforts were made to keep the tissue samples cool (4°C) or frozen as soon as possible after collection prior to transport to the Southeastern Cooperative Wildlife Disease Study (SCWDS, University of Georgia, Athens, GA) for testing. Once the samples arrived at SCWDS, they were stored at -20°C until testing was performed. After the tissues were thawed, and prior to DNA extraction, they were grossly examined and any samples of poor post-mortem condition (based on gross appearance and odor) were discarded.

Harvest date and location (county, state) were recorded for all samples, as well as the age and sex of the turkey if they were determined. Age and sex determinations were based on a combination of morphological characteristics, including plumage, presence/absence of spurs, and/or tarsometatarsus measurements [25]. For comparison, previously reported data from asymptomatic wild turkey samples from South Carolina [11] were included in this analysis.

To further evaluate the most appropriate tissue for LPDV testing in wild turkeys, samples of liver, spleen, and bone marrow were each collected from 15 LPDV-positive hunter-harvested wild turkeys and 20 LPDV-positive wild turkey diagnostic cases that were submitted to the SCWDS. Birds were designated as “positive” if LPDV proviral DNA was detected in one of the three tissues using the procedures described below. All of the LPDV-positive wild turkey diagnostic cases included in this analysis were found moribund or dead, but none had gross or microscopic evidence of lymphoproliferative disease.

### Testing Procedures

Tissue samples were stored at SCWDS at -20°C until testing was performed. Samples were thawed once and processed during the same day. Prior to processing each individual tissue sample in the laboratory, all instruments utilized were first disinfected using Microban Germicidal Cleaner (Coraopolis, PA, USA) followed by bead sterilization at 240° C for 5 minutes. Bone marrow was extracted by fracturing the tibiotarsus with rongeurs and removing a sample of marrow with sterilized forceps. Liver and spleen samples were collected using sterile scalpel blades and forceps.

DNA was extracted from liver, spleen, and bone marrow samples using the Qiagen DNeasy Blood and Tissue Kit (Qiagen, CA, USA). Extracted samples were tested for LPDV proviral DNA using published protocols and primers targeting a portion (431nt) of the *gag* polyprotein (partial p31/partial CA) [11]. Stringent protocols and controls were utilized in all PCR assays to prevent and detect contamination. DNA extraction, amplification, and product analysis were performed in separate dedicated laboratory areas. A subset of LPDV-positive samples (n = 126) were confirmed by sequencing (using the same primers that were used for amplification) of the p31/CA PCR product and subsequently used in phylogenetic analysis. Newly obtained p31/CA sequences have been deposited into GenBank under the accessions KP299678—KP299804.

### Data Analysis

Results from each state were analyzed by placing a 95% confidence interval around each prevalence estimate. Chi-square test with Yate’s correction was used to compare differences among different age and sex classes and geographic regions. Due to small sample sizes, fisher’s exact test was used to compare differences between different tissues tested from diagnostic cases or turkeys harvested by hunters.

### Phylogenetic Analysis

We performed a phylogenetic analysis on 184 partial p31/capsid (CA) sequences of all LPDV strains recovered from wild turkeys in the USA during the period 2009–2013 (58 previously published sequences and 126 newly obtained here), combined with the prototype Israeli strain recovered in 1978. These sequences were aligned in MUSCLE [26] which resulted in a final data set of 185 sequences, 413 nt in length. A maximum likelihood (ML) phylogeny was then estimated using PhyML 3.0 [27] and employing the GTR+Gamma+Proportion Invariant (GTR+G+I) model of nucleotide substitution, with all parameter values estimated from the data in hand, and utilizing a combination of nearest neighbor interchange (NNI) and subtree pruning and regrafting (SPR) branch-swapping. To infer support for individual nodes, a bootstrap analysis was conducted using 1000 replicate ML trees, using the nucleotide substitution model specified above and NNI branch-swapping.

To determine the extent of phylogeographic structure in the data—that is, whether there is more phylogenetic clustering by US state of sampling than expected by chance alone—we employed two phylogeny-trait association tests: the Association Index (AI) and Parsimony Score (PS), both of which are available in the BaTS package [28]. In addition, we used the Maximum Clade (MC) statistic to determine which individual US states showed the strongest spatial clustering. Phylogenetic uncertainty in the data was incorporated through the use of the posterior distribution of trees determined from a Bayesian Markov chain Monte Carlo analysis undertaken using the BEAST package [29]. For this analysis we employed the GTR+G+I substitution model as above, a constant population size prior, and a relaxed uncorrelated lognormal molecular clock, which was run for 100 million generations. Finally, we performed 1000 random permutations of sampling locations to create a null distribution for each BaTS statistic.

## Results

In total, 564 of 1,164 (47%) wild turkey samples were positive for LPDV proviral DNA (Table 1). Wild turkeys in all 17 states had a relatively high prevalence of LPDV (Fig 1). State-wide prevalence rates ranged from 26% in Oklahoma to 83% in New Hampshire (Table 1); however, there was significant overlap of confidence intervals for most individual states. When analyzed by geographic region, LPDV prevalence in Northeastern states was significantly higher than in Mid-Atlantic (χ^2^ = 33.991, p<0.0001) and Southeast (χ^2^ = 31.258, p<0.0001) states and the prevalence in the Central states was significantly lower than the other three regions (Northeast: χ^2^ = 52.007, p<0.0001; Mid-Atlantic: χ^2^ = 9.790, p = 0.0018; Southeast χ^2^ = 12.007, p = 0.0005) (Table 2). When only states that submitted bone marrow samples were included in the analyses (12 states; n = 653), similar regional differences were noted except the Central states were no longer significantly lower than the Southeast states (χ^2^ = 2.206, p = 0.1374) (Table 2). Of the 1,164 turkeys included in this study, age and sex were determined on 1,042 (89.5%) of the samples. Overall, adult turkeys had a significantly higher prevalence of LPDV than juveniles (χ^2^ = 21.342, p<0.0001), although there was no significant difference in prevalence between males and females (χ^2^ = 0.01, p = 0.9200) (Table 3).

**Table 1:** Prevalence of LPDV proviral DNA from hunter-harvested turkey samples from various states.

State	Tissue type	n	# Positive (% w/ 95% CI)
South Carolina	Liver	74	33 (45% ±11%)
West Virginia	Liver	47	26 (55% ±14%)
New York	Liver	7	4 (57%)
	Bone Marrow	266	128 (48%)
	TOTAL	273	132 (48% ±6%)
Virginia	Bone Marrow	59	17 (29% ±12%)
Florida	Liver	171	77 (45% ±7%)
Louisiana	Liver	96	57 (59% ±9.8%)
Oklahoma	Liver	27	7 (26% ±17%)
New Jersey	Bone Marrow	48	22 (46% ±14%)
Missouri	Liver	39	14 (36%)
	Bone Marrow	35	8 (23%)
	TOTAL	74	22 (30% ±10%)
Georgia	Liver	34	12 (35%)
	Bone Marrow	14	8 (57%)
	TOTAL	48	20 (42% ±14%)
New Hampshire	Bone Marrow	30	25 (83% ±13%)
Vermont	Bone Marrow	28	20 (71% ±17%)
Kansas	Liver	5	1 (20%)
	Bone Marrow	18	7 (39%)
	TOTAL	23	8 (35% ±19%)
Massachusetts	Bone Marrow	9	5 (56% ±33%)
Maine	Bone Marrow	61	50 (82% ±10%)
Rhode Island	Bone Marrow	9	3 (33% ±31%)
North Carolina	Liver	7	3 (43%)
	Bone Marrow	76	30 (39%)
	Spleen	4	1 (25%)
	TOTAL	87	34 (39% ±10%)
**TOTAL**		**1164**	**564 (47%)**

**Table 2:** Regional differences in prevalence of LPDV proviral DNA among hunter-harvested turkeys.

Tissue type	Region*	n	No. positive (%)**
Combined tissues	Northeast	137	103 (75.2)^a^
	Mid-Atlantic	427	197 (46.1)^b^
	Southeast	476	227 (47.7)^b^
	Central	124	37 (23)^c^
Bone marrow	Northeast	137	103 (75.2)^a^
	Mid-Atlantic	373	167 (44.8)^b^
	Southeast	90	38 (42)^b,c^
	Central	53	15 (28)^c^

*States in each region: Northeast (Maine, Vermont, and New Hampshire), MidAtlantic (New York, New Jersey, Virginia, West Virginia), Southeast (Georgia, Louisiana, South Carolina, Florida, North Carolina), and Central (Oklahoma, Missouri, Kansas)

**Significant differences (p<0.05) between regions within each of the two groups [combined tissues and bone marrow] using a chi-square test are indicated by different letters.

**Table 3:** Differences in prevalence of LPDV proviral DNA in wild turkeys between different age and sex categories across all 17 states sampled.

	Positive / Number tested (% positive)	
	Juvenile	Adult
Male	73/213 (34%)	327/640 (51%)
Female	33/85 (39%)	57/104 (55%)

Age and sex was not determined on 122 of the turkeys included in this study.

The ability to detect LPDV proviral DNA in different tissues varied between wild turkeys that were hunter-harvested and those that were submitted as diagnostic cases (Table 4). Overall, bone marrow had the highest level of detection of LPDV proviral DNA. However, there was no significant difference in LPDV detection between liver, spleen, and bone marrow collected from wild turkey diagnostic cases (p>0.1), whereas detection in hunter-harvested turkeys was significantly higher in bone marrow relative to spleen (p = 0.0349) or liver (p = 0.0063).

**Table 4:** Results of paired tissue testing of liver, spleen, and bone marrow samples collected from wild turkeys that were diagnostic cases or harvested by hunters.

Tissue Tested	Positive / Number tested	
	Diagnostic cases	Hunter-harvested
Liver	19/20^a^	8/15^a^
Spleen	16/20^a^	9/13^a^
Bone Marrow	20/20^a^	15/15^b^

*Significant differences (p<0.05) between tissue types within each group [diagnostic cases or hunter-harvested turkeys] using a fisher’s exact test are indicated by different letters.

**Fig 2:**
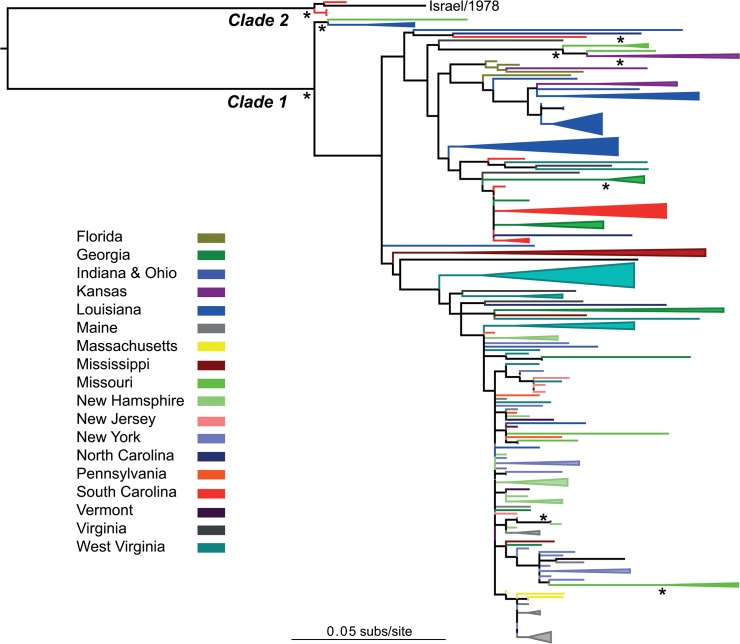
Phylogenetic relationships of LPDV sampled from wild turkeys in the United States of America. Sequences are color-coded according to the US state of isolation (see key), with viral sequences from the same state that form monophyletic groups “collapsed” and shown as triangles (the size of the triangle is a function of the number of sequences in the group). All branch lengths are scaled according to the number of nucleotide substitutions per site, and the tree is mid-point rooted for clarity only. Bootstrap support values >70% are shown by the * symbol.

The phylogenetic analysis of 185 wild turkey LPDV sequences revealed the presence of the two major viral clades defined previously (Fig 2 and S1 Fig) [11]. However, it is striking that aside from four sequences obtained from hunter-harvested wild turkeys in South Carolina during 2011, no other viruses from Clade 2, which contains the original Israeli prototype strain, have been detected in the USA suggesting that this lineage has experienced limited onward transmission. In contrast, Clade 1 viruses have spread across the eastern USA, although some state-specific clustering is observed (which is discussed below). In addition, Clade 1 contains three divergent members (3/LA/2013, 6/LA/2012 and 205/MO/2012) that comprise a distinct lineage.

As was apparent from the overall phylogeny, our phylogeographic analysis revealed a relatively strong clustering by state (n = 21) across the tree as a whole (p = 0 for both the AI and PS clustering statistics, and manifest as “collapsed” monophyletic groups in Fig 2), although with clear examples of geographic movement as noted by the mixing of states within the tree. The analysis of the strength of clustering in individual states was hampered by the relatively small numbers of sequences from each locality, although the strongest clustering (p < 0.001) was observed in Georgia, Florida, Louisiana, Maine, Mississippi, New York, South Carolina, and West Virginia.

## Discussion

Currently, there is very little information on avian retroviruses in wild turkeys. In 2009, the first reported case of LPDV in both a wild (non-domestic) bird and in North America was identified in a wild turkey in Arkansas diagnosed with lymphoproliferative disease [11]. Including this index case, over the next three years LPDV was identified in 41 wild turkey diagnostic cases from 18 states, of which only a small minority (n = 6) had evidence of lymphoproliferative disease. The results of this current study on reportedly asymptomatic hunter-harvested wild turkeys indicate that LPDV is geographically widespread throughout the eastern USA. Collectively, these data suggest that LPDV infection in wild turkeys is common, but associated viral-induced neoplastic disease is rare. Currently, the mechanism of LPDV-induced oncogenesis is unknown.

Consistent with previous surveillance results [11], a relatively high prevalence of LPDV was identified in wild turkeys from each sampled state. Regionally, the Northeastern states had the highest prevalence, followed by the Mid-Atlantic and Southeastern states, and finally the Central states. Explanatory variables for these regional differences in prevalence are unknown at this time, but could relate to multiple factors associated with the host, environment, or pathogen, including wild turkey population densities, habitat availability, artificial movement of infected birds, or interactions among different wild turkey flocks. Although variability in sample collection and storage techniques could impact these results, there were no obvious procedural differences between states or regions (other than what was described in the methods). When these data are combined with Allison et al. [11], the known distribution of LPDV in wild turkeys extends from Maine to Florida and as far west as Colorado. Additional surveillance is needed to better understand the prevalence and geographic distribution of LPDV in North American wild turkey populations, particularly focusing on: 1) smaller spatial scales to further define prevalence and identify whether “hot spots” of activity may exist, and 2) Canada, Mexico, and Central and Western states where sampling to date has been limited or non-existent.

There are six recognized subspecies of wild turkeys in the North America. The most widespread subspecies is the Eastern wild turkey which extends throughout the eastern part of North America from southern Canada to northern Florida [30]. In addition, this subspecies has been the source for many translocation efforts throughout the USA and Canada [31]. In this study, genetic testing was not performed to delineate the subspecies of wild turkey that were sampled; however, the geographic area in which surveillance was conducted encompasses the reported range of three different subspecies, including the Eastern (predominate subspecies overall in most sampled states), Osceola (predominate subspecies in central and southern Florida) and Rio Grande (predominate subspecies in central and western Oklahoma and Kansas). While the data presented herein do not allow for any conclusions on the relative susceptibility of these three subspecies to LPDV, differences may in fact exist based on previous studies in domestic turkeys. Experimentally, four strains of domestic turkeys varied in their susceptibility to LPDV based on incidence of lymphoproliferative disease, which ranged from 4 to 36% of the inoculated group [15]. To date, the reasons or mechanisms for the observed differences are unknown. It also is unknown whether similar genetically-controlled differences in susceptibility to LPDV exist between subspecies of wild turkeys, or whether such variability would be apparent when evaluating susceptibility based on viral infection (as reported herein) rather than occurrence of lymphoproliferative disease.

No difference in prevalence of LPDV was noted between sexes; however, adult wild turkeys were significantly more likely to be positive than juveniles. This is consistent with a previous study on LPDV in domestic turkeys, in which the age of bird at time of viral exposure was a significant factor in determining susceptibility. Experimentally, domestic turkey poults challenged with LPDV at 4-weeks-old experienced a higher incidence of lymphoproliferative disease than poults challenged at 1-day-old, which were more likely to develop a viremia in the absence of overt disease [15]. Although the reasons for the age-related differences in susceptibility were not determined, plausible explanations include protective effects of maternal immunity in 1-day-old poults and/or requirements of a mature and competent immune system for development of lymphoproliferative disease. It is currently unknown why the adult wild turkeys in this study had a higher prevalence of LPDV than juveniles. All of the juvenile wild turkeys were of sufficient age to have a competent immune system and no longer be experiencing the protective effects of maternal immunity. Considering the prolonged viremia of LPDV in domestic turkeys [12], which can persist for 8–10 months, it is possible that the higher prevalence in adult birds is simply associated with increased age and subsequently longer period for virus exposure. To date, surveillance for LPDV has not been conducted in wild turkey poults. This represents a critical gap in understanding LPDV ecology and defining potential population impacts. While overt disease associated with LPDV infection appears to be rare in older juvenile and adult wild turkeys, it is possible that higher levels of mortality could occur in younger poults. Such morbidity or mortality would be extremely difficult to detect through routine wildlife disease monitoring programs, as clinically-ill or dead poults do not last long on the landscape due to predation and scavenging, and rarely are submitted for post-mortem examination (Justin Brown, *personnel communication*).

When spleen, liver, and bone marrow samples collected from the same wild turkeys were compared, bone marrow was the most effective tissue for detection of LPDV proviral DNA. However, the relative success of LPDV detection in the three tissues was dependent on whether the samples were collected from wild turkeys that were diagnostic cases or harvested by a hunter. There was no significant difference in LPDV detection between liver, bone marrow, or spleen collected from diagnostic specimens, whereas, bone marrow was significantly more sensitive than the other two tissues when samples were collected from hunter-harvested wild turkeys. The relative sensitivity of the three tissues is consistent with the existing data on LPDV pathobiology. In domestic turkeys, LPDV infection preferentially targets lymphoid tissues, with replication occurring first in the bone marrow and then virus disseminating to other lymphoid organs including the spleen, thymus, and bursa of Fabricius [32]. Differences in LPDV detection between sample types (i.e., diagnostic or hunter-harvest) may also relate to how the tissues were collected and stored prior to arriving at the laboratory. To ensure the best possible post-mortem condition of the tissues, diagnostic specimens are typically cooled down as soon as they are found and every effort is made to maintain the cold chain and deliver the specimen to the laboratory as soon as possible. Although efforts were made in this study to keep hunter-harvested turkey samples cool or frozen until processing, these sample types were more likely to experience a delay in chilling of the tissues and/or temperature fluctuations during storage and transport from the field. An alternate explanation is that diagnostic specimens had a higher concentration of LPDV proviral DNA in the tested tissues. Although none of the diagnostic specimens tested in this study had evidence of lymphoproliferative disease, it is possible that the clinically-ill birds had higher viral loads that predisposed them to another disease or had higher viral loads in the tissues due to another primary disease process.

To date, there have been no reports of LPDV in domestic turkeys or other poultry in North America; however, as the virus was just recently identified in this continent, surveillance efforts have likely been minimal. Natural infections with LPDV have only been reported in domestic and wild turkeys. Experimentally, chickens were a permissive host for LPDV and developed lymphoproliferative disease, but ducks and geese were not susceptible [33]. While the wide geographic distribution and high prevalence of LPDV in wild turkeys would suggest that this virus represents a disease threat for poultry, an accurate risk assessment is currently difficult due to the limited knowledge on the ecology, epidemiology, and pathobiology of LPDV in North American galliforms. Two critical pieces of information that are needed in order to begin to define the risk of LPDV for poultry include understanding the potential routes of LPDV transmission within and between wild turkeys and poultry, and determining whether current genetic strains of poultry present in North America are susceptible to the New World viral strains.

While it appears that LPDV is endemic in wild turkeys in the eastern USA and an occasional source of neoplastic disease, little is known about this virus. To date, attempts to culture LPDV have failed [12] and this has significantly limited research efforts, including challenge studies. In addition to improving diagnostic tools for LPDV detection, further research is needed to define the epidemiology, modes of transmission, and pathogenesis in wild turkeys; determine the impacts of LPDV on wild turkey populations; and assess the risks of LPDV to domestic turkeys, including commercial and backyard flocks.

## Supporting Information

S1 Fig Phylogenetic relationships of LPDV sampled from wild turkeys in the United States showing the individual strains. All viruses in the tree are designated as identification number/state of collection/year of sampling. As in Fig 2, monophyletic groups that are collapsed are shown as triangles. All branch lengths are scaled according to the number of nucleotide substitutions per site, and the tree is mid-point rooted for clarity only. Bootstrap support values >70% are shown with an asterisk (*) symbol.
(EPS)
